# Neurofibrosarcoma of the gallbladder: a case report

**DOI:** 10.1186/1477-7819-11-189

**Published:** 2013-08-12

**Authors:** Xiao-Fang Liu, Kun Tang, Lu-Lu Sui, Gang Xu

**Affiliations:** 1 Department of Hepatobiliary Surgery, Affiliated Yantai Yuhuangding Hospital, Qingdao University Medical College, Yantai 264000, China

**Keywords:** Gallbladder, Neurofibrosarcoma

## Abstract

Sarcoma of the gallbladder is a rare entity. This report presents an extremely rare clinical case of a neurofibrosarcoma of the gallbladder. On examination, a mass was felt in the right hypochondrium. An ultrasound of the abdomen showed a mass in the gallbladder. Computed tomography (CT) scan of the abdomen showed a grossly distended gallbladder with soft tissue mass in the gallbladder. The mass was diagnosed as carcinoma of the gallbladder and an extended cholecystectomy was performed. Histopathological examination revealed spindle-cell proliferation and the possibility of a malignant tumor of mesenchymal origin.

## Background

Neurofibrosarcoma of the gallbladder is a rare form of tumor compared to adenocarcinoma and other sarcomatous tumors of the gallbladder [1]. Here, we report a case of a 72-year-old female patient with neurofibrosarcoma of the gallbladder who underwent an extended cholecystectomy. This is the first report of neurofibrosarcoma of the gallbladder in English and non-English literature.

## Case presentation

A 72-year-old female patient had been hospitalized for three days with pain in the right upper quadrant of the abdominal region. History reveals that the patient had experienced spontaneous pain, which increased after eating. The patient did not feel abdominal distension, nausea, vomiting and fever. Physical examination revealed body temperature (TPR) was 36.5 to 37.3C°; the heart rate (HR) of the patient was 78 beats/min and blood pressure (BP) was 120/90 mmHg. In addition, skin and tunica dura of the patient were not stained yellow; superficial lymphoid node was not swollen; abdomen was flat; GI form and peristaltic wave(−) subcutaneous varicose vein of abdominal wall(−) and abdominal muscle was soft. The patient felt light tenderness in the right upper quadrant without rebound tenderness and muscle tonus. Tumescent gallbladder could be palpable and was about 6×6 cm with Murphy sign(+). Liver and spleen were not touched below costal margin. Bowel sound (BS) was 2 to 3 min.

### Auxiliary examination

**Figure 1:**
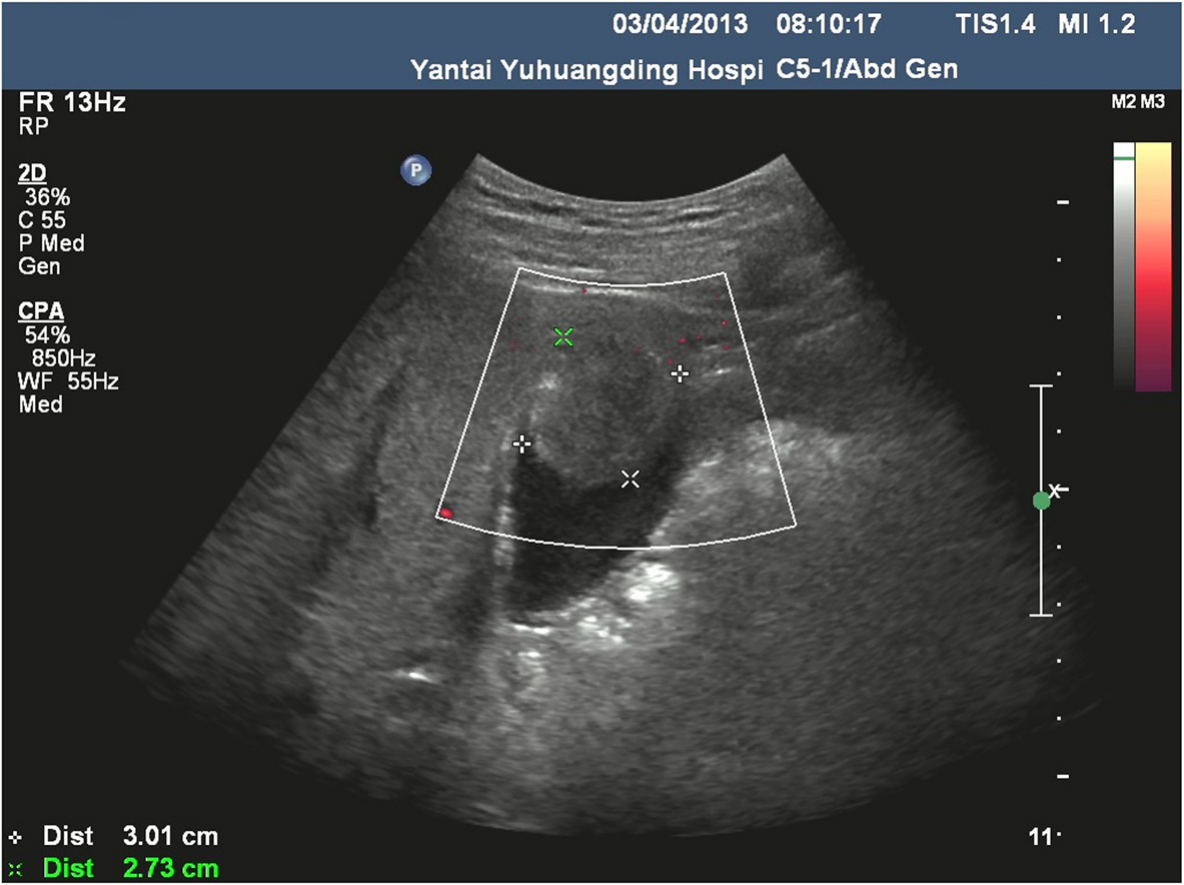
Ultrasonography B (Ultra-B) showed increasing volume of gallbladder, thicker wall and tumor.

**Figure 2:**
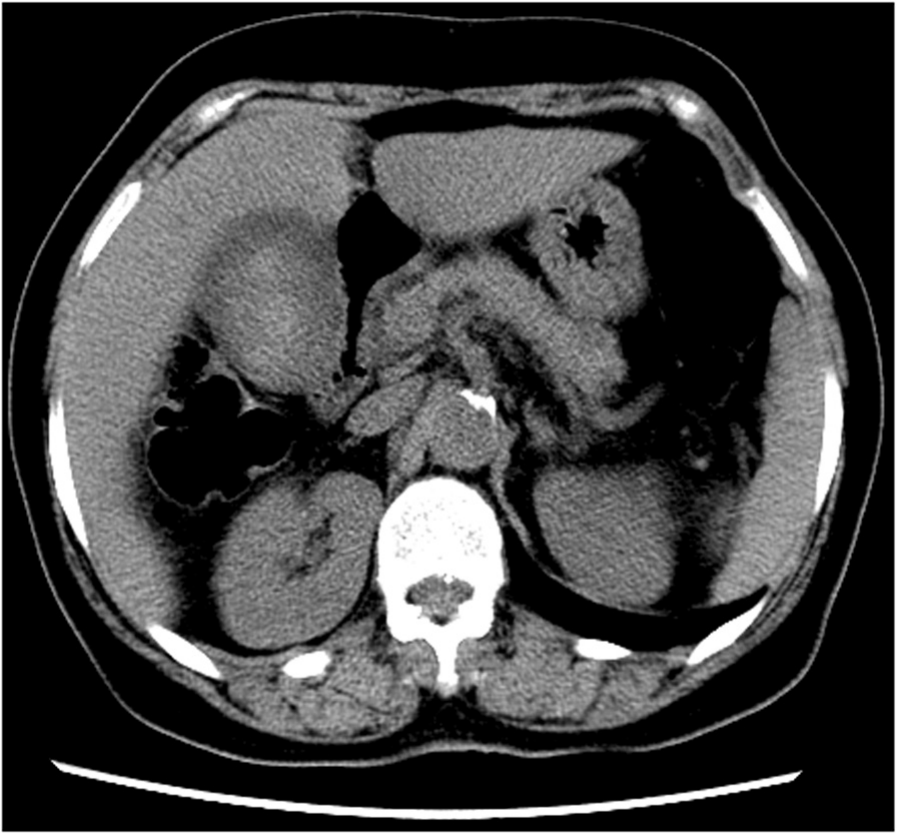
Plain computed tomography (CT) abdomen showing gallbladder grossly distended and soft tissue mass in the gallbladder.

**Figure 3:**
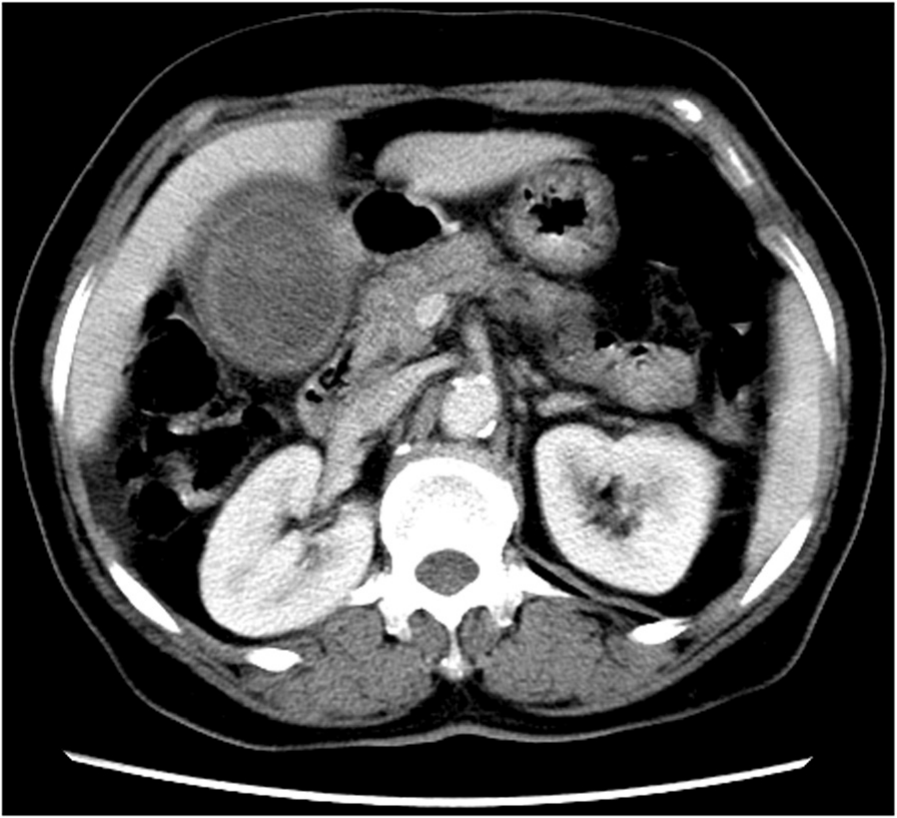
Enhancement computed tomography (CT) showed soft tissue tumor and manifest intensification.

Blood counts and liver function were normal. Carbohydrate antigen (CA)19-9, carcinoembryonic antigen (CEA) and α-fetoprotein (AFP) were normal. Ultrasonography B (Ultra-B) showed increased volume of gallbladder, thickening of wall (0.6 cm) and a 6.4×4.9 cm of tumor (6.4×4.9 cm) could be viewed (Figure 1). Plain computed tomography (CT) scan of the liver, gallbladder and pancreas showed increased volume of gallbladder (Figure 2). Soft tissue tumor could be seen and manifestation intensified after enhancement (CT value 72 Hu). The bile duct of the left hepatic lobe was found to be slightly expanded. The size and shape were normal. The ratio of liver lobe and segment was normal. The density of liver parenchyma was well-distributed with specific mass (Figure 3).

### Findings during surgical procedure

The liver of the patient showed chronic liver disease alteration but no metabasis nodus. The size of the gallbladder was 12×10×6 cm. The color was gray-red. The capsule wall of the gallbladder was thickened without being touched by the tumor on the plasma membrane. The tumor extended up to the visceral surface of the bottom of the gallbladder. The size was about 8×6 cm with hard texture and less movement. There were no metabasis nodus and swelling of lymphoid nodes on ligament hepatoduodenale, stomach, small intestine, pelvic cavity, abdominal membrane and root of mesentery. The surgical intervention included resection of the gallbladder, partial resection of the fifth segment of the liver and skeletonized dissection of the ligament hepatoduodenale.

### Histopathological examination

**Figure 4:**
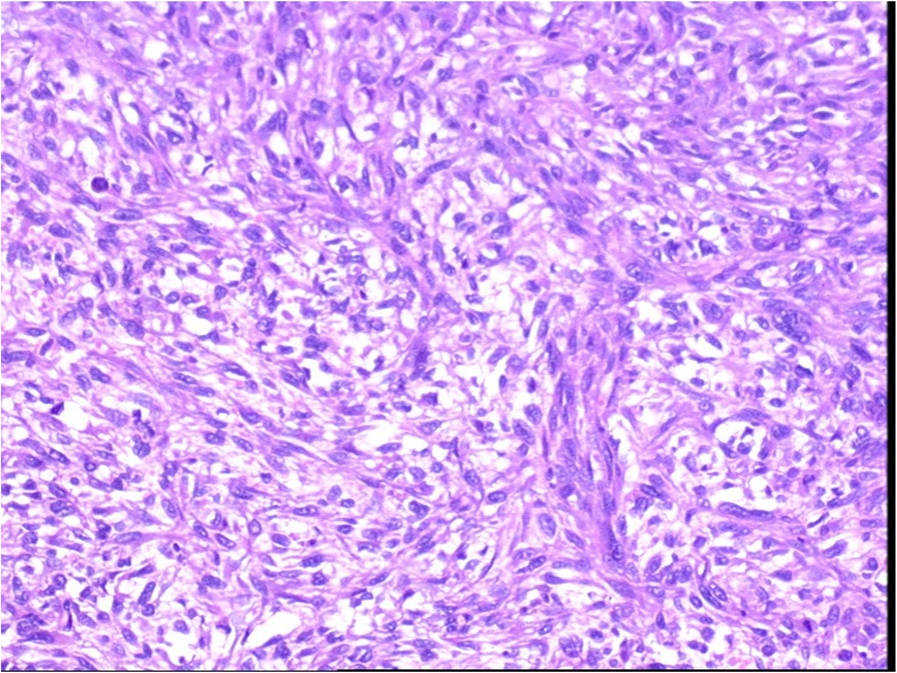
**Photomicrograph of the tumor showing the neoplastic cell makr-fusiform shape.** The karyotin of the cell had few nucleoli, light-red cytoplasm, and arranged fasciculation, weaving appearance and plaliform (hematoxylin and eosin (H&E) ×200).

A gray-red tumor 7 × 4.5 cm in size was removed from the gallbladder. Microscopic examination revealed that neoplastic cells were makr-fusiform shape, few short-fusiform shapes and similar-round shape. The karyotin of the cells was well-distributed, contained few nucleoli, and had light-red cytoplasm with slender cellosilk. The cells were arranged with a fasciculated and weaving appearance. The nucleo-heterology of the cells was quiet, but neoplastic cells were abundant and intensive. The nuclear fission could be found 1 per 2 to 3 high power field (Figure 4). The lymphoid nodes of 12a (hepatic artery nearby), 12b (bile duct nearby), 12c (portal vein nearby), 8 group (hepatic common artery nearby), 13 group (after head of pancreas nearby) showed no neoplastic cells.

### Immunohistochemistry

**Figure 5:**
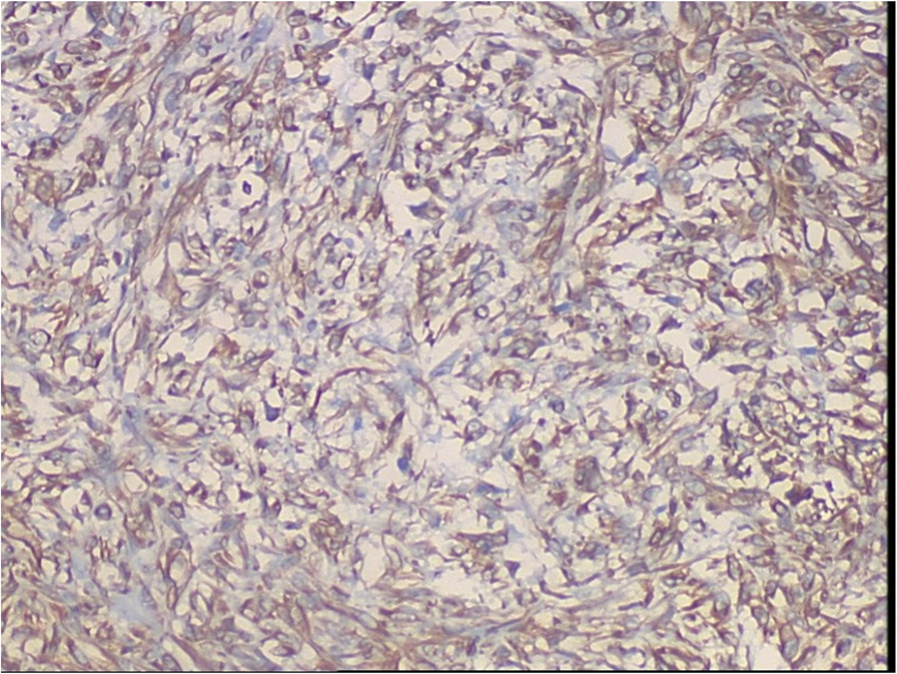
Photomicrograph of immunostained section using vimentin showing cytoplasmic positivity in almost all the tumors cells (SP, streptavidin-perosidase, ×200).

**Figure 6:**
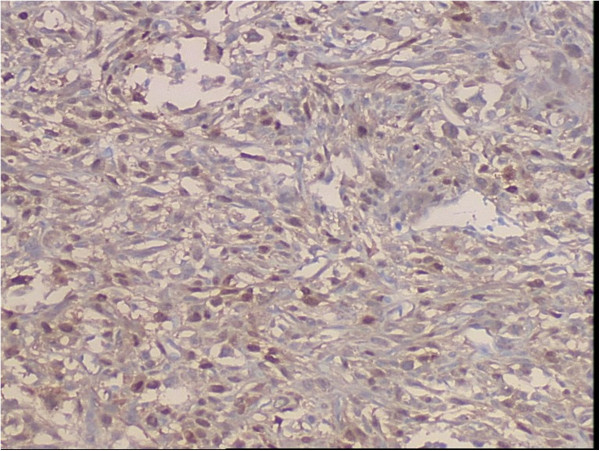
Photomicrograph of immunostained section using S100 protein showing cytoplasmic and matrix positivity in the tumors cells (SP, streptavidin-perosidase, ×200).

**Figure 7:**
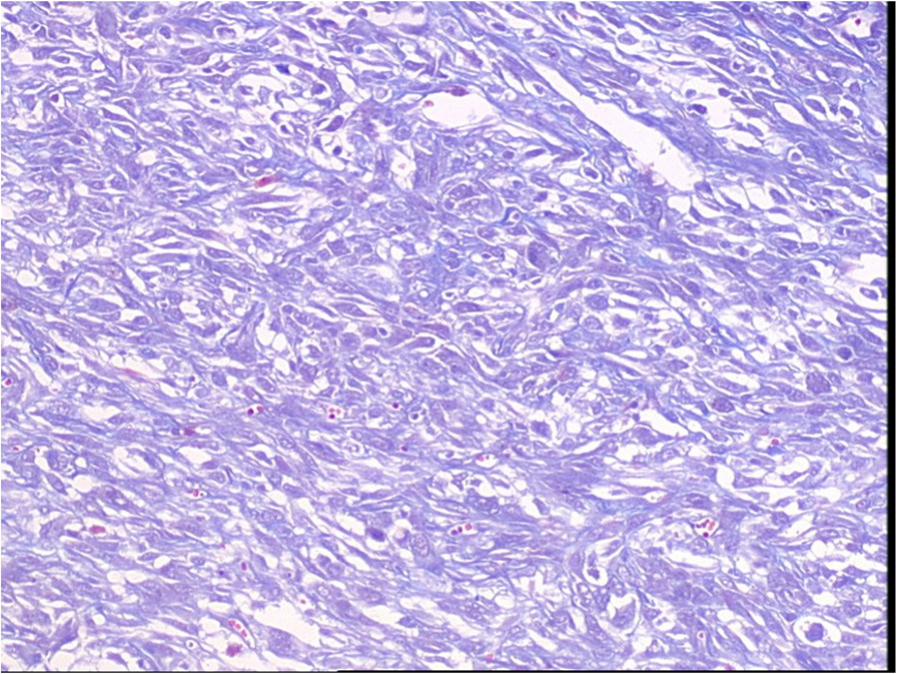
Photomicrograph of immunostained section showing Masson dyed:blue in the most of tumors cells (×200).

Vimentin (++), S-100 (+), CD34 (+), Neuron specific enolase (+), CK (−), CD68 (−), CD163 (−), CD31 (−), SMA (−), Masson dyed:blue (Figures 5, 6 and 7)

## Discussion

Among malignant tumors of the gallbladder, adenocarcinoma accounts for 82%, followed by undifferentiated carcinoma, which accounts for 7%, squamous cell carcinoma for 3%, gland scale mixed cancer for 1% and other uncommon malignant tumors such as lymphatic sarcoma, rhabdomyosarcoma, reticulum cell sarcoma, fibrosarcoma, carcinoid tumor and carcinoma sarcoma [1]. Most of neurofibrosarcoma originates from peripheral nerves. The tumor is often seen in patients between the ages of 30 to 50 years. The location of tumor usually occurred in the trunk, followed by retroperitoneal, head and neck [2]. In our report, neurofibrosarcoma occurred in the gallbladder. It has not been reported previously. The condition is difficult to diagnose preoperatively and can be easily misdiagnosed as gallbladder cancer.

The mass assessed by abdominal ultrasound, CT and/or magnetic resonance imaging (MRI) revealed a huge soft tissue tumor. Laboratory detection of tumor markers, such as AFP, CA19-9 and CEA were usually negative. Patients were treated using multimodal approaches including surgery, chemotherapy and radiotherapy, based on the time of diagnosis. Surgical intervention is the main treatment strategy. Radiotherapy was considered in patients with risk of local recurrence due to micro- or macroscopically incomplete resection. Adjuvant chemotherapy was not administered routinely, but only in some cases with metastasis. Commonly used drugs were doxorubicin, vincristine and cyclophosphamide. The disease could present with lymphatic and hematogenous metastasis. Pretreatment investigations included chest x-ray or chest CT scan, and whole-body bone scan. A 5-year survival rate was about 30%. In this case, the patient has not received any adjuvant therapy so far (after over 6months of follow-up) and there is no evidence of local recurrence and distant metastasis.

## Conclusion

This report presents for the first time, an extremely rare case of a patient with neurofibrosarcoma of the gallbladder. The condition was managed by an extended cholecystectomy. The case described was a clinical entity that could have been easily misdiagnosed as gallbladder cancer. We think competent imaging and predictive immunological studies could improve the diagnosis of this disease. Moreover, improved surgery and newer methods of adjunctive and neoadjunctive treatment would result in better prognosis for patients with this tumor.

## Consent

Written informed consent was obtained from the patient for publication of this report and any accompanying images.

## Abbreviations

AFP: α-Fetoprotein; BP: Blood pressure; BS: Bowel sound; CA19-9: Carbohydrate antigen (CA)19-9; CEA: Carcinoembryonic antigen; CT: Computed tomography; HR: Heart rate; MRI: Magnetic resonance imaging; TPR: Body temperature; Ultra-B: Ultrasonography B.

## Competing interests

The authors declare that they have no competing interests.

## Authors’ contributions

LXF performed the literature review. TK did the immunohistochemistry. All authors read and approved the final version of the manuscript.
